# When Common Presentations Conceal Rare Diagnoses: A Case Report of Splenic Actinomycosis

**DOI:** 10.7759/cureus.109838

**Published:** 2026-05-28

**Authors:** Christopher Blanton, Lynde Tucker, Alec Wallace, Colin Danko

**Affiliations:** 1 Department of Emergency Medicine, University of Texas Southwestern Medical Center, Dallas, USA

**Keywords:** actinomyces, anchoring bias, cognitive bias, diagnostic challenge, infectious disease, splenic abscess

## Abstract

Splenic abscess secondary to Actinomyces species is an exceedingly rare condition that presents with a constellation of nonspecific symptoms often leading to delayed presentation, diagnostic uncertainty, and misidentification. While Actinomyces is typically an indolent infection, splenic abscess has a high mortality when untreated due to the risk of splenic rupture.

This case report highlights a rare instance of isolated Actinomyces splenic abscess in a 52-year-old man with a history of intravenous drug use, hepatitis C, and recent incarceration who presented with several weeks of hemoptysis, fevers, night sweats, pleuritic chest pain, and left upper quadrant abdominal pain. The history and clinical presentation were most concerning for malignancy, tuberculosis, or other pulmonary infectious etiologies. Computed tomography imaging revealed a 14.9 x 7.7 x 12.1 cm splenic abscess with associated pleural effusion. A percutaneous drain was placed by interventional radiology, and the resulting fluid culture grew *Actinomyces meyeri*. In consultation with infectious disease specialists, prolonged antibiotic therapy was started with four weeks of intravenous piperacillin-tazobactam followed by six months of oral amoxicillin.

This case highlights both the diagnostic challenge of splenic actinomycosis and the importance of maintaining a broad differential diagnosis to avoid anchoring bias or early diagnostic closure in high-risk patients with nonspecific symptoms.

## Introduction

Actinomyces infections of the abdomen are uncommon but potentially life-threatening conditions. The Actinomyces genus comprises Gram-positive, anaerobic rods that form branching filaments and are commensal organisms of the mucus membranes, gastrointestinal tract, and the female genital tract. Splenic abscesses are themselves rare, and involvement by Actinomyces species is exceedingly uncommon, accounting for less than 1% of reported cases [1,2]. These infections often present a diagnostic challenge due to their indolent course, nonspecific symptoms, and delayed clinical presentation. Patients may exhibit fevers, abdominal pain, and constitutional symptoms frequently related to local inflammation or involvement of adjacent structures [1,3].

Splenic abscesses most commonly arise from hematogenous spread, trauma, or immunosuppression and are associated with significant morbidity and mortality, particularly when diagnosis is delayed. Cases of splenic Actinomyces are often associated with diagnostic delays, with reports suggesting an average delay of up to two months [3,4]. This atypical etiology further complicates recognition in the emergency department (ED), where early identification is critical to improving patient outcomes.

We present the case of a 52-year-old man with a high-risk medical and social history who initially presented with symptoms concerning for tuberculosis or malignancy and was ultimately diagnosed with a splenic abscess caused by Actinomyces. His clinical presentation, in the context of the above risk factors, initially directed the diagnostic focus away from the abdomen. This case underscores the importance of maintaining a broad differential diagnosis in the ED, as well as the risk of anchoring bias and premature diagnostic closure in complex presentations. It also highlights key clinical features and diagnostic considerations, including early imaging and consideration of occult intra-abdominal sources in patients with systemic symptoms, relevant to emergency physicians encountering this rare but serious condition.

## Case presentation

This patient is a 52-year-old man with a pertinent history of intravenous drug use, opiate use disorder on suboxone, treated hepatitis C, mood disorder, and recent incarceration, who currently resides in a group home, who presented with several weeks of progressive cough with blood-tinged sputum, subjective fevers, generalized malaise, drenching night sweats, and left-sided pleuritic chest pain. He additionally complained of some left upper quadrant (LUQ) abdominal discomfort. Two weeks before this presentation, he was seen in a clinic where there was concern for tuberculosis, but at the time of his ED presentation, he had not followed up for his lab work yet. A recent chest X-ray was reportedly normal per the outpatient note, and he stated that while he was incarcerated, he tested negative for tuberculosis.

On arrival at the ED, he was febrile to 100.8°F but otherwise hemodynamically stable. Examination of his abdomen revealed tenderness to palpation in the LUQ with guarding and mild discomfort with palpation throughout the remainder of the abdomen. No rebound or costovertebral angle tenderness was present, and his cardiopulmonary exam was unremarkable.

Given his clinical presentation, including hemoptysis, night sweats, a high-risk social history such as incarceration, intravenous drug use, and hepatitis C, as well as the development of fever in the ED, a broad differential diagnosis was considered. With his systemic symptoms, malignancy was high on the differential. Likewise, with his history of incarceration, night sweats, and hemoptysis, tuberculosis needed to be evaluated for. Additionally, infectious etiologies such as endocarditis with possible septic emboli were considered, particularly in the context of intravenous drug use history. Pneumonia and other infectious etiologies were included in the differential diagnosis. Given the high-risk history and high suspicion for malignancy or pulmonary pathology, a computed tomography (CT) scan of the chest was obtained. However, the presence of LUQ tenderness on examination prompted expansion of the differential to include intra-abdominal sources of infection, leading to further evaluation with CT of the abdomen and pelvis.

Initial laboratory studies demonstrated leukocytosis of 16 × 10³/µL with neutrophilic predominance, including an absolute neutrophil count of 12.1 × 10³/µL, prompting blood cultures and initiation of empiric broad-spectrum antibiotics. Lactate was normal at 0.7 mmol/L. Renal and hepatic function were preserved, with a creatinine of 0.88 mg/dL, blood urea nitrogen of 9 mg/dL, aspartate aminotransferase of 26 U/L, and alanine aminotransferase of 14 U/L.

CT of the abdomen and pelvis demonstrated a large subcapsular splenic fluid collection measuring 14.9 × 7.7 × 12.1 cm with associated mass effect on adjacent structures and a small focus of internal air, consistent with a splenic abscess (Figures 1, 2). CT of the chest additionally demonstrated a moderate left-sided pleural effusion (Figures 3, 4).

**Figure 1:**
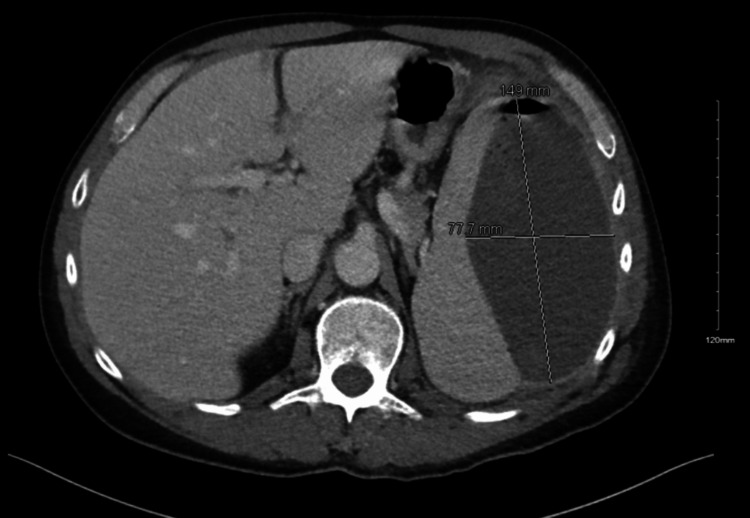
Axial CT of the abdomen and pelvis without contrast demonstrating 14.9 x 7.77 cm splenic fluid collection CT: computed tomography

**Figure 2:**
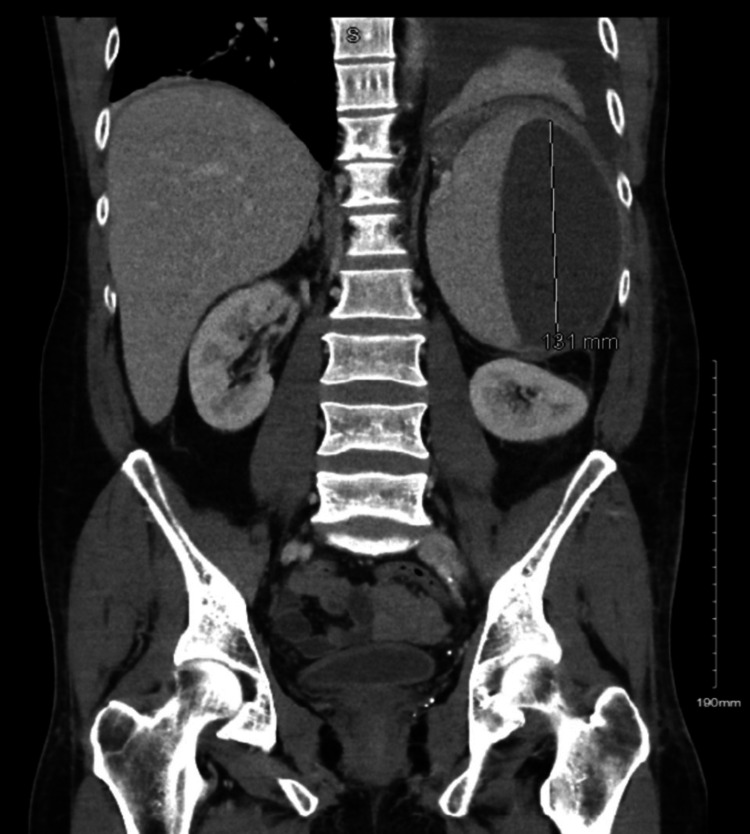
Coronal CT of the abdomen and pelvis without contrast, redemonstrating splenic fluid collection CT: computed tomography

**Figure 3:**
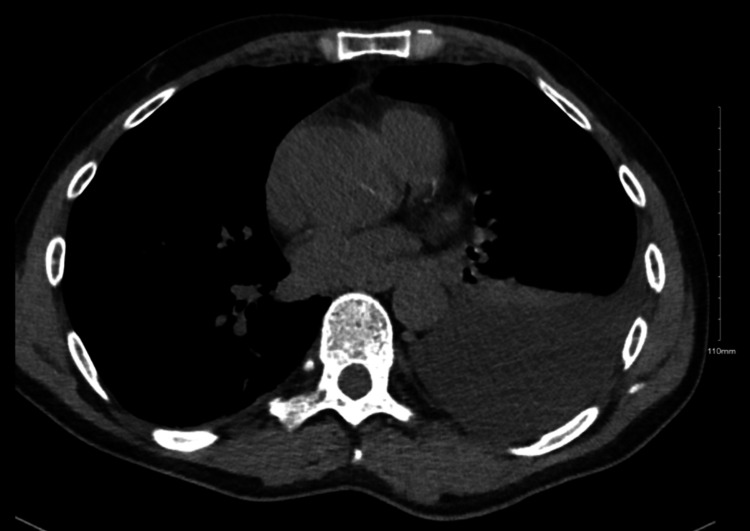
Axial CT of the chest with contrast, redemonstrating left-sided moderate pleural effusion CT: computed tomography

**Figure 4:**
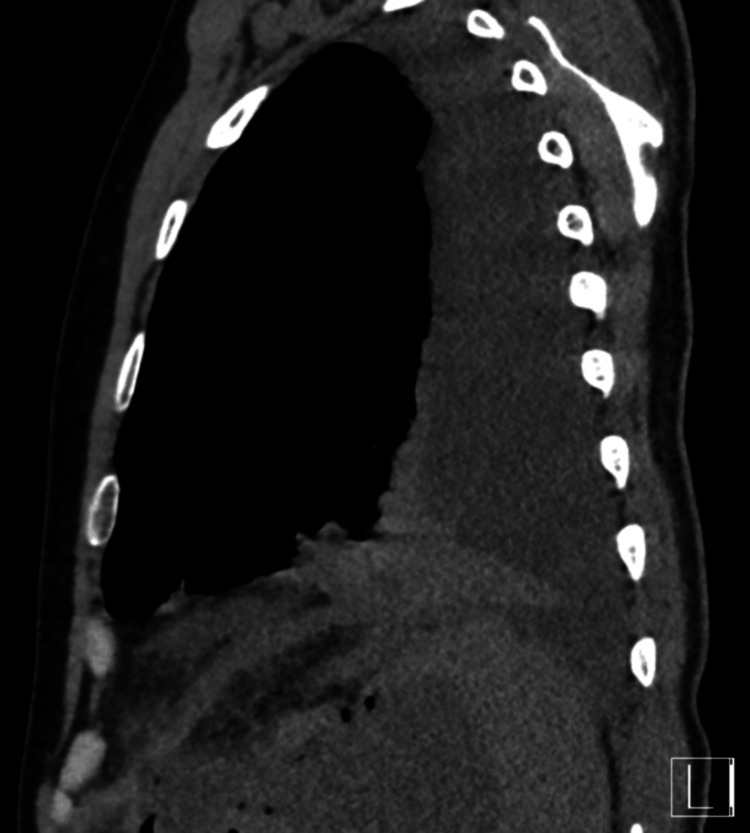
Sagittal CT chest with contrast, redemonstrating splenic fluid collection and the presence of a moderate left pleural effusion CT: computed tomography

The patient was admitted to the hospital medicine service under airborne isolation while under investigation for tuberculosis. During hospitalization, a thoracentesis was performed, yielding 1,200 mL of amber-colored pleural fluid, which was consistent with an exudative effusion by Light’s criteria (serum protein 7.0 g/dL; pleural fluid protein 4.3 g/dL). Pleural fluid analysis demonstrated a pH of 7.66 and a lactate dehydrogenase level of 229 U/L. Cytology revealed abundant neutrophils and macrophages with a total nucleated cell count of 4,065 cells/µL (59% neutrophils) and 2,000 red blood cells, without evidence of malignant cells. Tuberculosis evaluation was negative, including two acid-fast bacillus cultures and a negative T-Spot test.

Interventional radiology was consulted for source control and placed a percutaneous drain into the splenic abscess, evacuating purulent, foul-smelling fluid. Aspirate cultures were sent for aerobic, anaerobic, fungal, and Nocardia testing. Initial Gram stain demonstrated abundant Gram-positive rods without mention of sulfur granules or branching filamentous forms. Anaerobic cultures grew *Actinomyces meyeri* on hospital day 4, with subsequent growth on aerobic cultures the following day. Fungal and Nocardia cultures remained negative.

Given the uncommon presentation of actinomycosis, infectious disease (ID) specialists recommended four weeks of intravenous piperacillin-tazobactam followed by six months of oral amoxicillin therapy. After a 12-day hospitalization, the patient was discharged to a skilled nursing facility with the splenic drain in place, and outpatient ID follow-up was arranged for ongoing treatment monitoring.

## Discussion

This case underscores the importance of maintaining a broad differential diagnosis and avoiding anchoring bias, particularly in patients with high-risk features. Although this patient's presentation of hemoptysis, fever, night sweats, and a history of recent incarceration raised early concern for tuberculosis or malignancy, premature diagnostic closure may have delayed the identification of this patient's rare infection.

This case describes a rare presentation of an isolated splenic abscess caused by *A. meyeri*. The diagnostic challenge is further emphasized by the nonspecific and variable nature of splenic abscess presentations, which can mimic other chronic diseases such as chronic granulomatous infections, malignant lesions, fungal infections, and inflammatory bowel disease [1,3]. The disease course can be indolent and is frequently complicated by diagnostic delays, with a symptomatic duration range of weeks to months. Reported symptoms include cough, fevers, anorexia, and weight loss [3,4]. A multicenter study examining 28 cases of actinomycotic infections across different anatomic locations noted that 92% of cases were not suspected at the time of admission [4]. Only one-third of patients have the classic triad of fever, LUQ pain, and splenomegaly [1]. In this patient’s case, the overlap of symptoms commonly associated with tuberculosis, pneumonia, or malignancy highlights how this diagnosis could have been overlooked. Furthermore, splenic abscess secondary to actinomycotic infection is exceedingly rare, with a reported frequency of 0.05%-0.7%, and splenic actinomycosis accounts for fewer than 1% of all actinomycotic infections, highlighting the rarity of this patient's presentation [1,2].

Actinomyces species are commensal organisms of the oral cavity, gastrointestinal tract, and female genital tract. Most infections arise from hematogenous dissemination or from contiguous spread from adjacent structures [4]. Infection typically requires disruption of mucosal barriers and is associated with risk factors such as loss of integrity of gut mucosa via trauma or infection, immunosuppression, prior transplant, HIV, chemotherapy, poor oral hygiene, alcoholism, or diabetes [5]. For this patient, ID had two hypotheses regarding the origin of this abscess. One potential explanation was that the abscess could have formed due to transient bacteremia of oral flora. Alternatively, the patient mentioned that he had suffered a strangulated hernia several months prior to his ED presentation, which could have led to a translocation event.

Diagnosis begins with maintaining a high suspicion. Imaging may demonstrate heterogeneous or multiloculated splenic fluid collections on ultrasound, CT, or magnetic resonance imaging, but definitive diagnosis requires histopathologic samples and cultures for confirmation, given the rarity of this infection [6]. Actinomyces can be detected by growth in an anaerobic culture. Histopathologically, the presence of sulfur granules in pus or histologic sections is suggestive of Actinomyces, but Nocardia can exhibit similar features as well [3]. Traditionally, splenic abscesses have been managed with splenectomy given the risk of splenic rupture, which is associated with high mortality. Percutaneous drainage and prolonged antibiotic therapy, given the high rate of antibiotic failure, are now considered effective alternatives in select patient populations and have the advantage of being less invasive [7]. Penicillin-class antibiotics remain the antibiotic of choice for the treatment of Actinomyces infections [7-9]. In our patient, percutaneous drain placement was performed given the size of the splenic abscess and the risk of splenic rupture, and a four-week course of IV piperacillin-tazobactam was followed by a six-month course of amoxicillin, given the extent of his infection.

Cases like this can lead to anchoring bias and premature closure among clinicians. Anchoring occurs when a clinician gives disproportionate weight to some features in a patient’s presentation. For example, in this case, this patient presented with fevers, night sweats, hemoptysis, and a history of incarceration, which initially raised concern for malignancy or tuberculosis. If we had anchored on this initial information, we might not have obtained abdominal imaging despite his exam also exhibiting LUQ abdominal pain. This may have led us to attribute his pleural effusion to another cause rather than revealing the true source of infection. Similarly, premature closure can occur when an initial working diagnosis appears to explain the clinical presentation well enough that diagnostic investigation is halted. Both anchoring and premature closure are known cognitive biases that affect emergency physicians. In the ED, these biases are intensified by the high-acuity, fast-paced environment where clinicians have multiple competing priorities at any given time. Clinicians can mitigate the risks of anchoring bias and premature closure by maintaining a broad differential, maintaining diagnostic flexibility, and maintaining awareness of possible bias during patient reassessment.

## Conclusions

An adult man with a high-risk medical and social history presented to the ED with multiple weeks of progressive symptoms concerning for an infectious vs. neoplastic respiratory pathology, leading to the risk of premature diagnostic closure and anchoring bias. A broad differential was considered, leading to imaging findings of a large splenic abscess that grew *A. meyeri* on culture. Definitive diagnosis and treatment required imaging, percutaneous drainage for source control with fluid cultures, and prolonged antibiotic therapy. Ultimately, the patient was successfully transitioned to a skilled nursing facility with his percutaneous drain in place for continued outpatient management. This case highlights the diagnostic complexity of this patient's splenic abscess from *A. meyeri*, a rare presentation with a reputation for symptomatic mimicry of other common diseases, which, if missed, carries a high risk of mortality due to splenic rupture. This case contributes to the limited literature on isolated splenic actinomycosis and underscores the importance of keeping a broad differential diagnosis in the ED to avoid the pitfalls of premature diagnostic closure and anchoring bias.
